# Supplementary material for: Estimating the extrinsic incubation period of malaria using a mechanistic model of sporogony
Source: PLoS Comput Biol. 2021 Feb 16;17(2):e1008658. doi: 10.1371/journal.pcbi.1008658 (PMC7909686; doi:10.1371/journal.pcbi.1008658)
Supplement: S1 Text — (DOCX) [file pcbi.1008658.s002.docx]

# Supporting Information

## Supplementary methods

### Mosquito survival (A(t))

To estimate the difference in mosquito survival due to infection status, $E$, we model the mosquito survival data using the Cox proportional hazards model as follows. The Kaplan-Meier survival curves of the mosquito survival data (S2 Fig) show that the proportion of mosquitoes alive does not decline exponentially indicating that the mosquito mortality rate is not constant. Indeed, a previous analysis of a subset of the data found the best fitting survival distribution to be the Gompertz, in which the mosquito mortality rate increases with age (i.e. there is senescence) [1]. The baseline hazard, $h_{0}(t)$, was modelled as Gompertzian:

$$\begin{aligned} h_{0}\left( t \right)=ae^{bt}. \#\left( 1.01 \right) \end{aligned}$$

The hazard, $h(t)$, is modelled accounting for differences in mosquito infection status, $E$ (= 1, if infected; = 0, if uninfected), and calculated as,

$$\begin{aligned} h\left( t \right)=h_{0}\left( t \right)e^{\beta_{E}E}, \#\left( 1.02 \right) \end{aligned}$$

where *β_E_* is a constant. The probability of being alive at time *t*, $A(t)$, is calculated by integrating the hazard to time t, as follows,

$$\begin{aligned} A\left( t \right)=e^{-\int_{0}^{t} h\left( t \right)dt}= e^{\frac{a}{b}\left( 1-e^{bt} \right)e^{\beta_{E}E}}. \#\left( 1.03 \right) \end{aligned}$$

As mosquitoes were removed for dissection or still alive at the end of the study some observations were right censored, that is we do not know the time when death would have occurred. For these observations, the likelihood is given by $A(t)$. The probability density function, $f(t)$, describing the event that death occurs at time *t*, for uncensored observations, is given by:

$$\begin{aligned} f\left( t \right)=h\left( t \right)A\left( t \right). \#\left( 1.04 \right) \end{aligned}$$

We suppose the infectious blood-fed group is a mix of infected and uninfected mosquitoes, meaning the true infection status of these mosquitoes was not known. The probability of an observable infection in this group is given by *δ* , but we cannot distinguish between mosquitoes that never received any parasites and those that cleared the infection. There may be a potential cost of immunity and tissue damage caused by the ookinete life stage, but given that the impact of malaria infection is still unclear [2], we assume that if the mosquito clears the malaria infection prior to the oocyst life stage, there is no additional mortality hazard due to infection. We account for uncertainty in the true infection status of mosquitoes by marginalising infection status out of the joint distribution as follows,

$$\begin{aligned} f\left( t \right)=\delta h_{0}\left( t \right)e^{\beta_{E}}e^{\frac{a}{b}\left( 1-e^{bt} \right)e^{\beta_{E}}}+ \left( 1-\delta\right)h_{0}\left( t \right)e^{\frac{a}{b}\left( 1-e^{bt} \right)}. \#\left( 1.05 \right) \end{aligned}$$

### Logistic growth model

The cumulative sporozoite prevalence at time $t$, $g(t)$, was estimated under the logistic growth model as,

$$g\left( t \right)= \frac{g_{max}}{1+e^{-c\left( t-t_{m} \right)}},$$

where $g_{max}$ is the upper asymptote, equivalent to the maximum possible sporozoite prevalence (used as an estimate of the human-to-mosquito transmission probability), $t_{m}$ and $c$ are constants that determine the growth rate [3]. The binary logistic model was fit to all mosquitoes that were dissected on or before the day the peak sporozoite prevalence was observed (binary coding; 1: sporozoites observed, 0: no sporozoites observed) simultaneously, using non-linear least squares.

References

1. Shapiro LLM, Whitehead SA, Thomas MB. Quantifying the effects of temperature on mosquito and parasite traits that determine the transmission potential of human malaria. PLOS Biol. 2017;15: e2003489.

2. Ferguson HM, Read AF. Why is the effect of malaria parasites on mosquito survival still unresolved? Trends Parasitol. 2002;18: 256–261. doi:10.1016/S1471-4922(02)02281-X

3. Paaijmans KP, Blanford S, Chan BHK, Thomas MB. Warmer temperatures reduce the vectorial capacity of malaria mosquitoes. Biol Lett. 2012;8: 465–468. doi:10.1098/rsbl.2011.1075
