# Supplementary material for: Estimating the extrinsic incubation period of malaria using a mechanistic model of sporogony
Source: PLoS Comput Biol. 2021 Feb 16;17(2):e1008658. doi: 10.1371/journal.pcbi.1008658 (PMC7909686; doi:10.1371/journal.pcbi.1008658)
Supplement: S1 Table — (DOCX) [file pcbi.1008658.s003.docx]

| **Parameter** | **Description** | **Prior parameters (subject to limits in next column)** | **Parameter limits** | **Justification of parameterisation (reference)** |
| --- | --- | --- | --- | --- |
| **Temperature-independent parameters** | | | | |
| *α_S _* | Shape parameter for the gamma distribution governing oocyst to sporozoite development time | $\alpha_{OS}\sim N(15.0, 2.5)$ | Lower: 0 | Yields a mean sporozoite development time of 8 days |
| *β_S_* | Rate parameter for the gamma distribution governing oocyst to sporozoite development time | $\beta_{OS}\sim N(1.875, 2.5)$ |  |  |
| *μ* | Mean number of G-stage parasites inoculating an infected mosquito | $\mu\sim N(3.0, 2.5$ | Lower: 0 Upper: 50 | The median and mean number of oocysts per mosquito has been found to be approximately 3 ^1–4^ |
| *k* | Overdispersion in the number of G-stage parasites inoculating an infected mosquito | $k\sim N(0.1, 2.5)$ | Lower: 0  Upper: 20 | The overdispersion of oocysts has been found to be below slightly below 0.1 ^3,4^ |
| *a* | Gompertz distribution parameter governing the baseline hazard | $a\sim N(0.05, 2.5)$ | Lower: 0  Upper: 5 | Assuming mosquito survival is solely determined by the baseline hazard, the mean prior values result in a median survival time of 10.3 days (0.5 – 30.9 days 95% survival quantiles) |
| *b* | Gompertz distribution parameter governing the baseline hazard | $b\sim N(0.05, 2.5)$ |  |  |
| *β__* | Cox proportional hazards model coefficient of infection status | $\beta_{E}\sim N(0.5, 1.0)$ | Lower: -5  Upper: 5 | The hazard odds ratio of infection is 1.65, given the mean prior value |
| *σ_δ_* | Standard deviation of the delta error term | $\sigma_{\delta}\sim N(0, 0.25)$ | Lower: 0 | There are no differences between experiments, given the mean prior values |
| *σ_survival_* | Standard deviation of the Cox model error term | $\sigma_{survival}\sim N(0, 0.25)$ |  |  |
| **Temperature-dependent parameters** | | | | |
| *a_GO_*, | Shape parameter for the gamma distribution governing G-stage parasite to oocyst development time | $\alpha_{IO}\sim N(15.0, 2.5)$ | Lower: 0 | At the prior mean value of *m_b_*(i.e. 0), temperature has no effect on the oocyst development rate. The shape and rate priors give a mean oocyst development time of 5 days |
| m*_β_* | Temperature-coefficient in eq (2.12) | $m_{\beta}\sim N(0, 2.5)$ | *m_b_* has no limits |  |
| *c_β_* | Intercept in eq (2.12) | $c_{\beta}\sim N(3.0, 2.5)$ | Lower: 0 |  |
| m_δ_ | Temperature-coefficient in eq (2.13) | $m_{\delta}\sim N(0, 2.5)$ |  | At the prior mean values, temperature has no effect on the transmission probability. Overall, these result in a prior mean transmission probability of 0.69 |
| c_δ_ | Intercept in eq (2.13) | $c_{\delta}\sim N(0.8, 2.5)$ |  |  |
| *β_C_* | Temperature coefficient in Cox proportional hazards model (eq (2.15) | $\beta_{C}\sim N(0.5, 1.0)$ | Lower: -5  Upper: 5 | Mosquitoes have a hazard odds ratio of 1.15 for  each 1°C increase in temperature (calculation accounts for temperature scaling) |

References

1 Pringle G. A quantitative study of naturally-acquired malaria infections in Anopheles gambiae and Anopheles funestus in a highly malarious area of East Africa. *Trans R Soc Trop Med Hyg* 1966; **60**: 626–32.

2 Rosenberg R, Andre RG, Somchit L. Highly efficient dry season transmission of malaria in Thailand. *Trans R Soc Trop Med Hyg* 1990; **84**: 22–8.

3 Taylor LH. Infection rates in, and the number of Plasmodium falciparum genotypes carried by Anopheles mosquitoes in Tanzania. *Ann Trop Med Parasitol* 1999; **93**: 659–62.

4 Billingsley PF, Medley GF, Charlwood D, Sinden RE. Relationship between prevalence and intensity of Plasmodium falciparum infection in natural populations of Anopheles mosquitoes. *Am J Trop Med Hyg* 1994; **51**: 260–70.
