# Supplementary figures and images for: Estimating the extrinsic incubation period of malaria using a mechanistic model of sporogony
Source: PLoS Comput Biol. 2021 Feb 16;17(2):e1008658. doi: 10.1371/journal.pcbi.1008658 (PMC7909686; doi:10.1371/journal.pcbi.1008658)

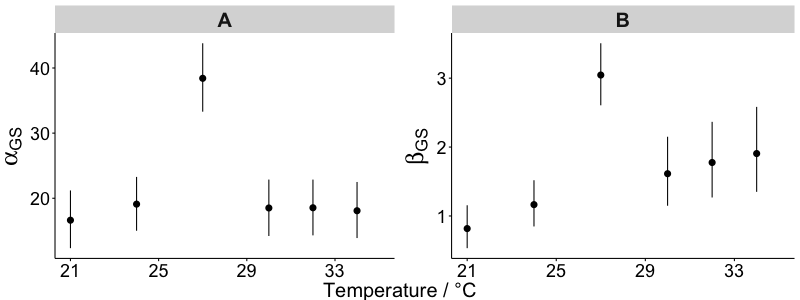

Supplement: S1 Fig — Panels show the posterior estimates of the two parameters (A: shape; B: rate) of the gamma distribution governing the development time between inoculation and observed sporozoites for the single temperature models. The posterior median and difference between the 2.5 and 97.5 posterior quantiles are represented by the points and vertical lines respectively. (TIF) [file pcbi.1008658.s006.tif]

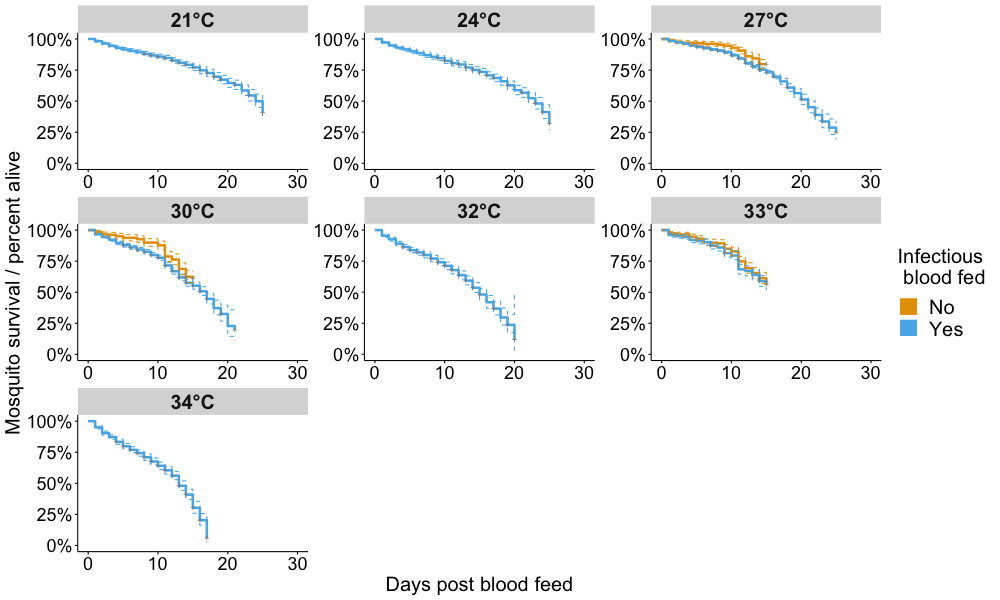

Supplement: S2 Fig — Mosquito survival data was obtained from three previously published studies [14,19,38]. Mosquitoes fed on infectious blood are shown in blue, mosquitoes fed on uninfected (control) blood are shown in yellow. (TIF) [file pcbi.1008658.s007.tif]

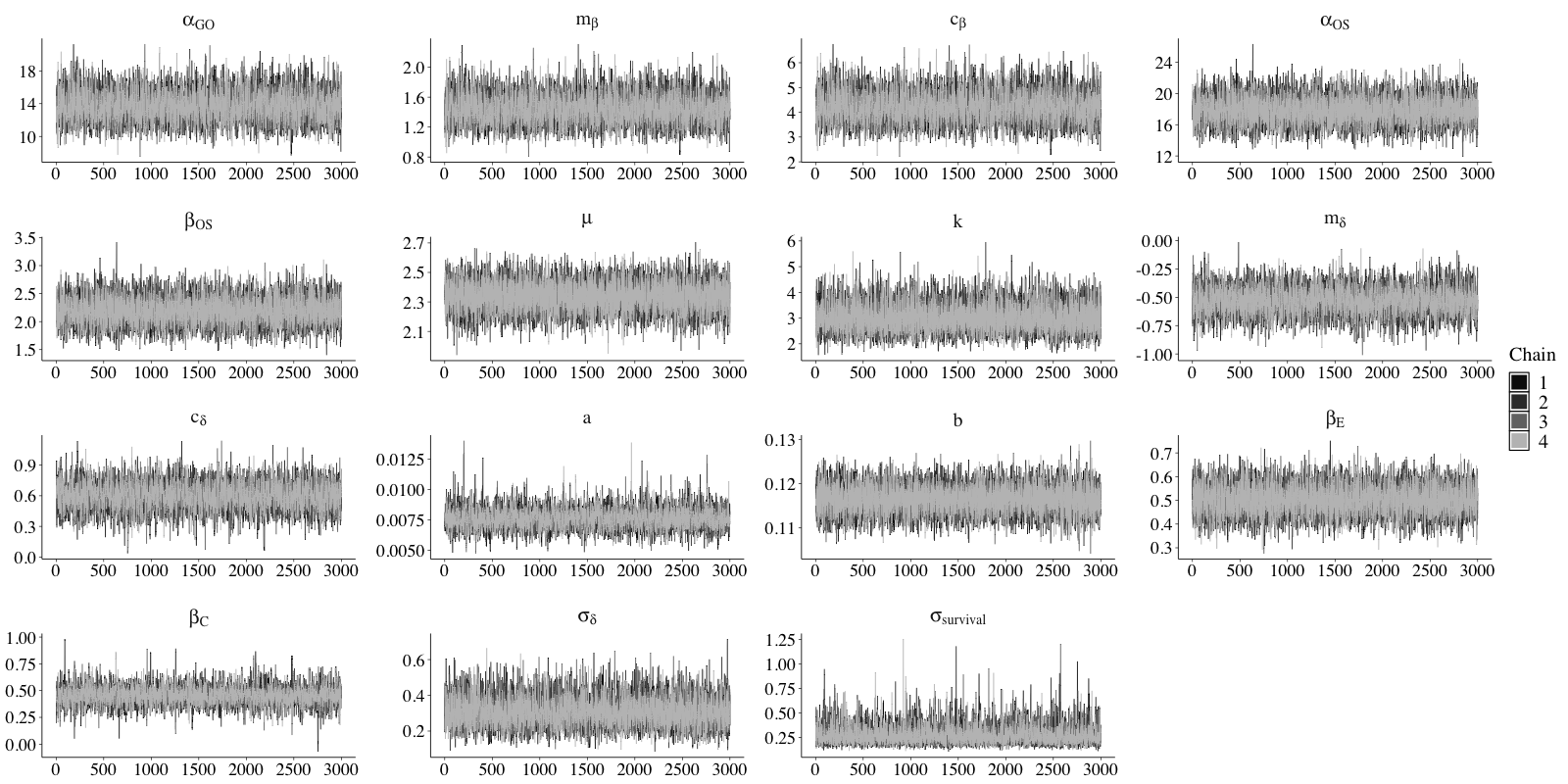

Supplement: S3 Fig — The horizontal and vertical axis give the iteration and parameter values respectively. Iterations during the warmup are not included. (TIF) [file pcbi.1008658.s008.tif]

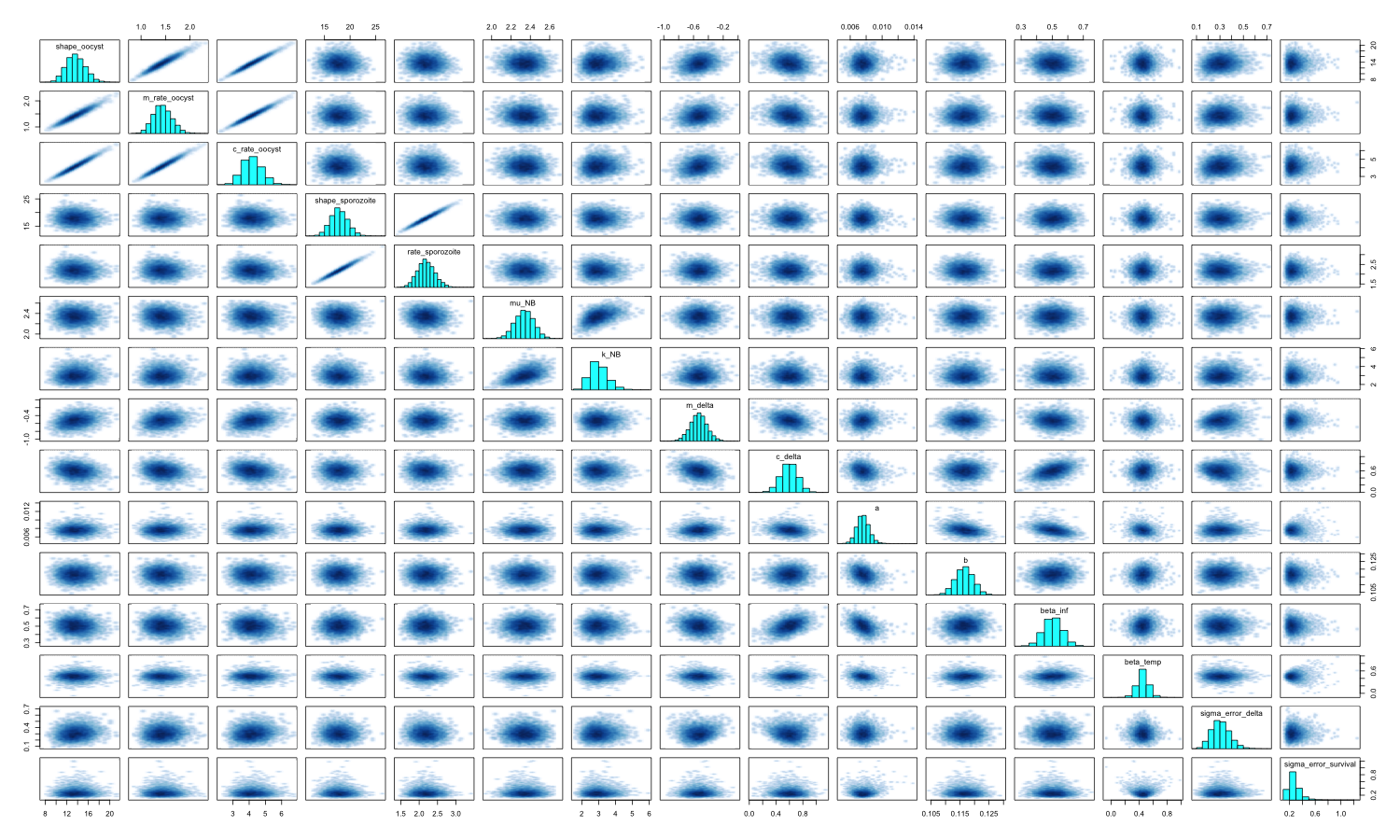

Supplement: S4 Fig — Parameter syntax is as follows: αGO (“shape_oocyst”), mβ (“m_rate_oocyst”), cβ (“c_rate_oocyst”), αOS (“shape_sporozoite”), βOS (“rate_sporozoite”), μ (“mu_NB”), k (“k_NB”), mδ (“m_delta”), cδ (“c_delta”), a (“a”), b (“b”), βE (“beta_inf”), βC (“beta_temp”), σδ (“sigma_error_delta”) and σsurvival (“sigma_error_survival”). (TIF) [file pcbi.1008658.s009.tif]

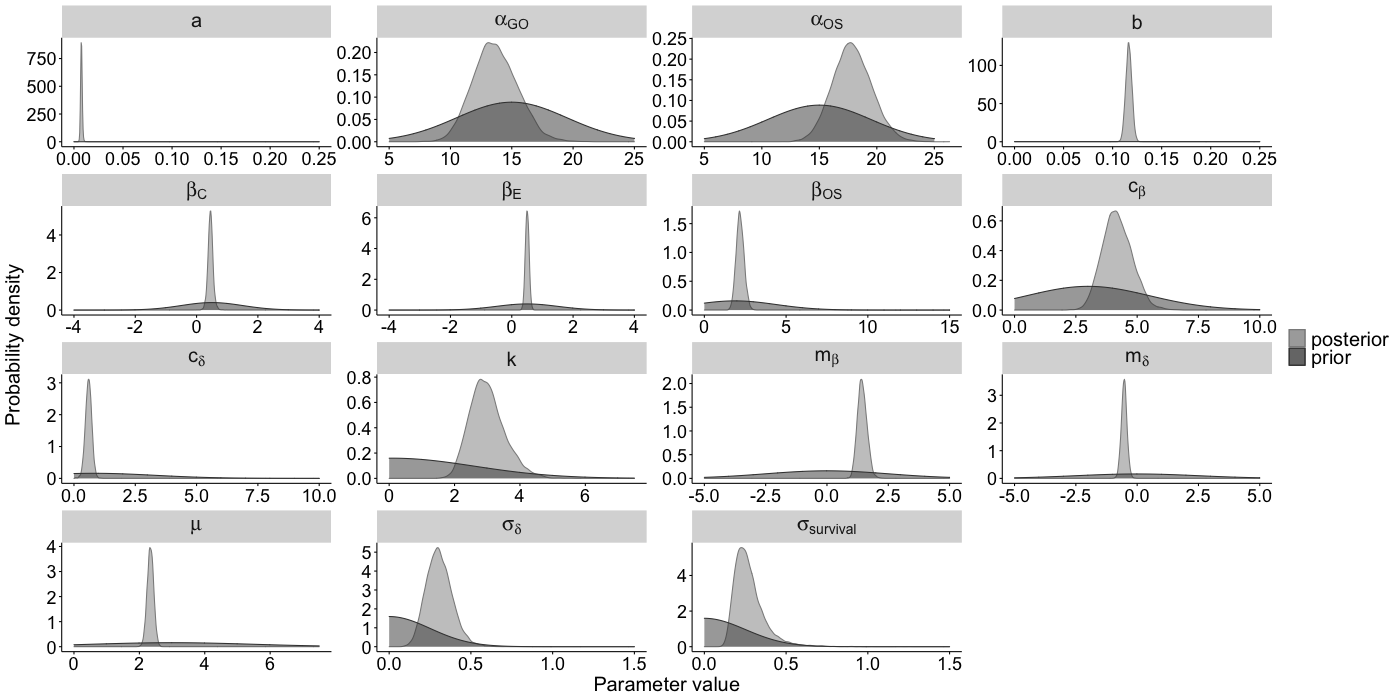

Supplement: S5 Fig — The posterior distributions are estimated by kernel density estimation with a gaussian smoothing kernel. (TIF) [file pcbi.1008658.s010.tif]

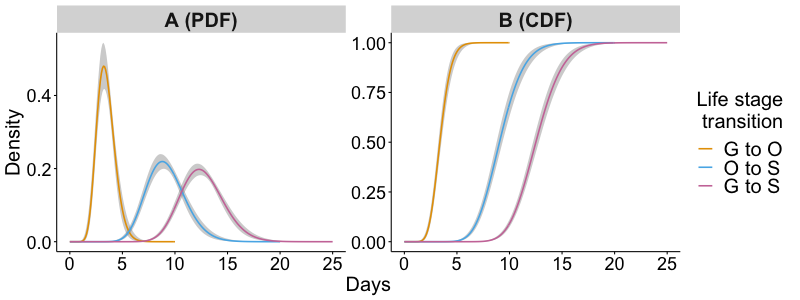

Supplement: S6 Fig — Panels A and B show the probability density function (PDF) and the cumulative probability density function (CDF) that an individual P. falciparum parasite within a mosquito (maintained under standard insectary conditions: 27°C) will transition from a given life stage to the next life stage at a given time post blood feed. The grey shaded area represents 2.5%-97.5% posterior quantiles of the estimated distributions. (TIF) [file pcbi.1008658.s011.tif]

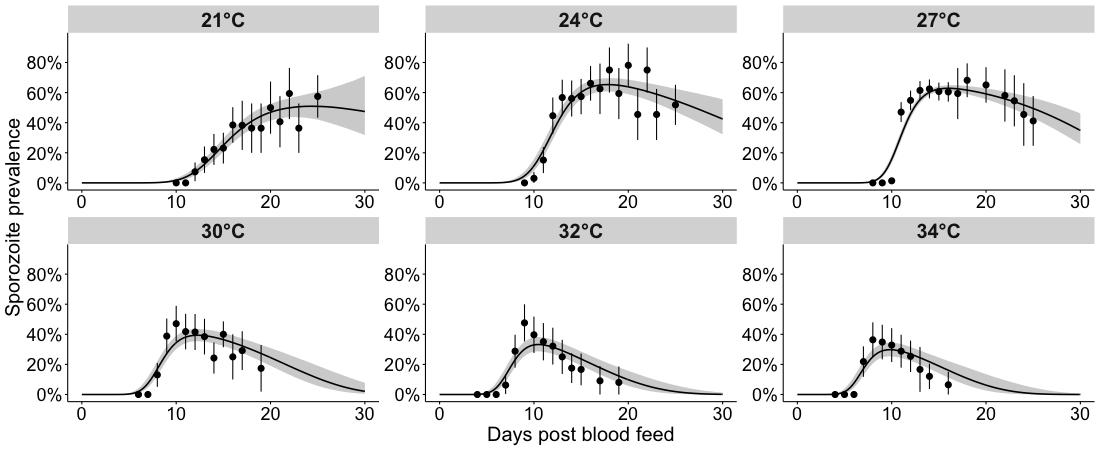

Supplement: S7 Fig — Black points: parasite prevalence of the laboratory mosquito data (95% binomial confidence intervals are given by the vertical black lines). The grey shaded area represents the 95% uncertainty intervals of the mean prevalence (posterior predictive means). The black line represents the median of the poster predictive means. (TIF) [file pcbi.1008658.s012.tif]

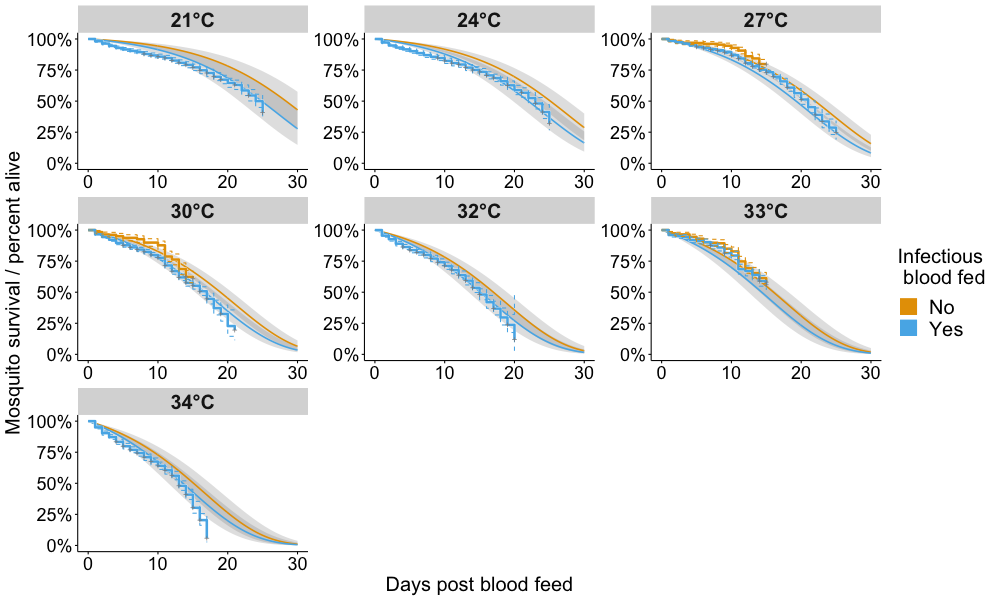

Supplement: S8 Fig — Kaplan Meier curves are stepped. The shaded area shows the 95% uncertainty intervals of the posterior predictive mean survival probability (A(t)) modelled by the Cox proportional hazards model. (TIF) [file pcbi.1008658.s013.tif]

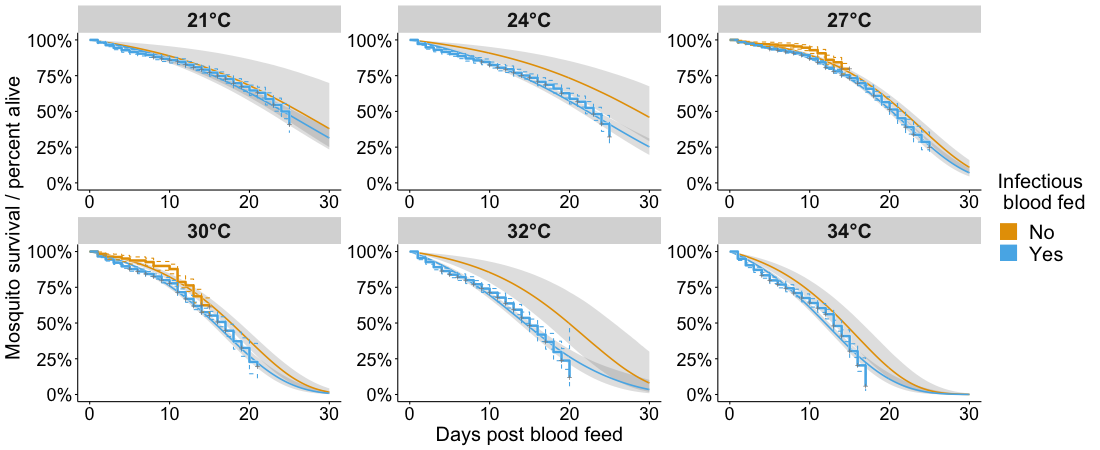

Supplement: S9 Fig — Kaplan Meier curves are stepped. The shaded area shows the 95% uncertainty intervals of the posterior predictive mean survival probability (A(t)) modelled by the Cox proportional hazards model. (TIF) [file pcbi.1008658.s014.tif]

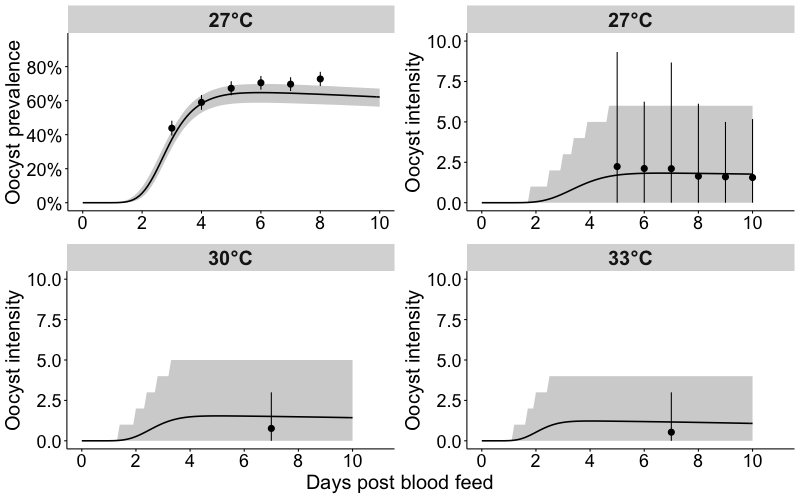

Supplement: S10 Fig — Oocyst prevalence plot: black points represent the prevalence of the laboratory mosquito data and the lines represent the 95% binomial confidence intervals. The grey shaded area represents the 95% uncertainty interval of the posterior predictive mean prevalence. Oocyst intensity plots: black points represent the mean oocyst intensity among all blood fed mosquitoes, the black line shows the posterior predictive mean oocyst count, and the grey shaded area represents the uncertainty in the mean oocyst count indicating the 2.5% and 97.5% negative binomial quantiles when fixing all parameters at their mean posterior values. (TIF) [file pcbi.1008658.s015.tif]

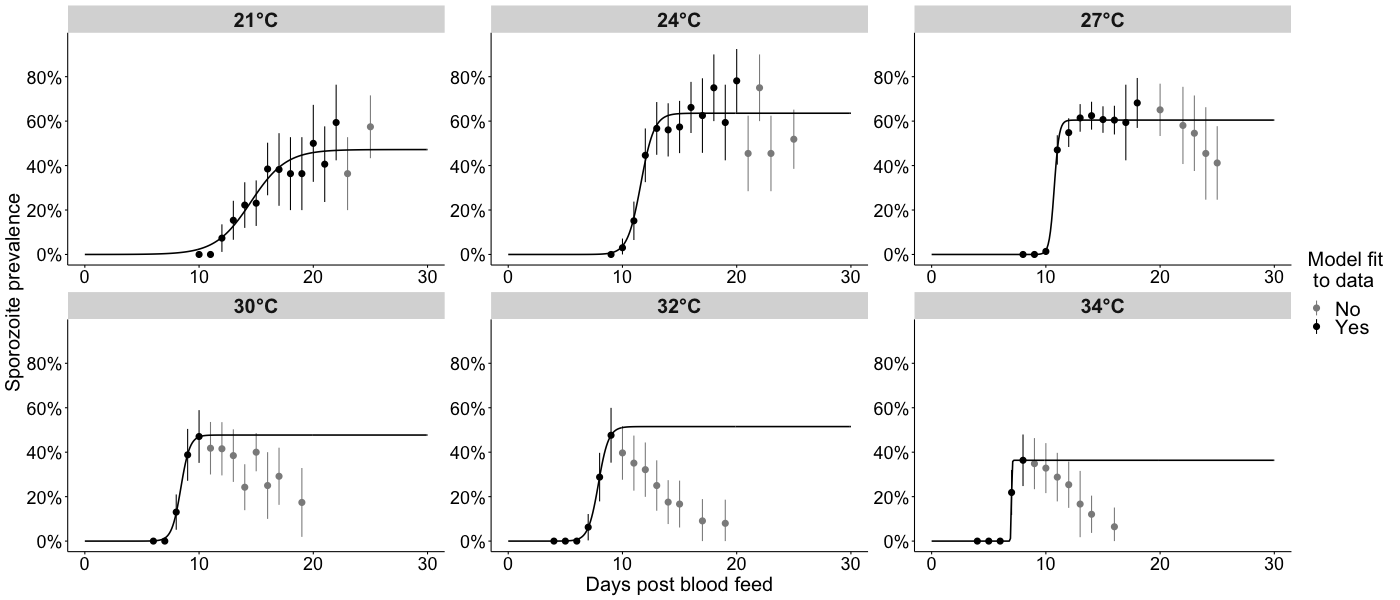

Supplement: S11 Fig — Logistic model fits (black line) to a subset of the sporozoite prevalence data (i.e. all data before observed peak sporozoite prevalence) are shown as solid lines; black points indicate those points were included in the fitting; grey points show those excluded. (TIF) [file pcbi.1008658.s016.tif]
