# Supplementary material for: Estimating the extrinsic incubation period of malaria using a mechanistic model of sporogony
Source: PLoS Comput Biol. 2021 Feb 16;17(2):e1008658. doi: 10.1371/journal.pcbi.1008658 (PMC7909686; doi:10.1371/journal.pcbi.1008658)
Supplement: Attachment — Submitted filename: PLOS Comp Bio response.docx [file pcbi.1008658.s017.docx]

Reviewer #1

In their paper, Stopard et al. model the time required for sporogony using a multiscale stochastic model and fit their model to experimental data from different studies. They compare their results to results obtained by using an existing model and find a greater variation in the extrinsic incubation period (EIP) using their model. The EIP is the duration of sporogony from uptake of parasites until infectious sporozoites can be found in the mosquito salivary glands. Overall, this reviewer thinks that this study addresses an important part of the malaria parasite life cycle, they use a model with greater flexibility than the predominantly (according to the authors) used logistic model, and their model has the potential to improve our understanding of the mosquito stage of malaria. However, some aspects of the modelling require further clarification. This reviewer’s expertise is not in the mosquito stage of malaria, thus it is difficult to assess the validity of Stopard et al.’s model assumptions or the significance and impact of their findings on the field of malaria parasite biology in mosquitos. However, regarding the mathematical model, the authors present a novel multiscale model of this biological process. At the parasite scale, they model the time until the parasite is in the next development stage as following a gamma distribution. At the mosquito scale, they take into account the human-to-mosquito transmission probability and death of mosquitos and at the observational level they model the fraction of infected mosquitos and the number of oocysts found in dissected mosquitos. The main results are the temporal dynamics of sporogony, the impact of temperature on the EIP from the model and the comparison of their model with a logistic model. Stopard et al. find a greater variation in the EIP than with a logistic model and a higher human-to-mosquito transmission probability. They argue that since estimates of transmission probability are highly sensitive to changes in EIP, a better understanding of the EIP is necessary to improve estimates of malaria transmission. Their methods appear to be technically sound and the authors clearly state their model assumptions and justify them. Nonetheless, questions regarding the logistic model, the comparison of the two models, and the what informs the heterogeneity in their model remain.

We thank the reviewer for the review and useful comments. We understand your concerns regarding further clarification of our model and comparisons between the logistic model and this approach, which we will hopefully address in our responses below and revisions to the manuscript.

Major comments:
1. Some aspects regarding the logistic model and the comparison of the two models are not clear to this reviewer. For example, why was a part of the data excluded? What were the criteria for excluding later observations? It appears that all data after the highest mean sporozoite prevalence was reached were excluded.

This is a good point, and we recognise that we did not previously make our reasons for fitting the logistic model which excluded later observations clear. This part of the manuscript was concerned with replicating a predominant approach used to estimate the EIP in the literature. This approach appears valid if sporozoite prevalence increases and plateaus over time. However, in the parasite-mosquito species combination investigated here the relationship is modal as infectious mosquitoes die at a faster rate. The previous method deals with this by fitting a logistic model to the upward trend in sporozoite prevalence data. It does this to avoid explicitly dealing with mechanisms leading to later declines in sporozoite prevalence, meaning observations after the peak sporozoite prevalence are removed. We agree, this approach has a number of flaws including, relying on the time when observed peak sporozoite prevalence occurs as an indicator of the time when all infected mosquitoes have developed sporozoites. Also, it does not explicitly model the biological mechanisms leading sporozoite prevalence to rise (and, potentially, fall). Because of these, we believe there is merit in developing a mechanistic model of sporogony, like the sort we present here.

To address this point, we have adapted lines 449 – 455 to,

“Given a decline in the observed sporozoite prevalence at later time points, the data was subset to include only data points before the time of peak observed sporozoite prevalence. This method has previously been used to enable the logistic function to be fit to modal data [14], but requires the following assumptions to be true: (1) the time when the observed sporozoite peak occurs in those mosquitoes sampled reflects the same attribute in the underlying population, and (2) all mosquitoes have developed sporozoites by the time observed sporozoite prevalence starts to decline”.

Given that the logistic model does not appear to include mosquito death, the higher human-to-mosquito transmission probability of Stopard et al.’s model does not seem surprising.

Yes, this is an astute observation: it seems reasonable that inclusion of mosquito death may partly explain this result. To this effect, we now include the following in the discussion lines 629 - 698,

“Nonetheless, accounting for potential differences in mortality is essential to avoid bias in parameter estimates: our estimates of the human-to-mosquito transmission probability, for example, were higher than those estimated by the logistic model”.

We do think this is an important result, however, since previous attempts to analyse these data did not account for this factor, which potentially could mean these previous estimates of the human-to-mosquito transmission probability were biased downwards. We have also added additional text at lines 568 to 572:

“At 27°C, for example, our single temperature model estimated an EIP_10_–EIP_90_ range of 9.2–13.7 days compared to 10.3–11.2 days estimated by the logistic approach. This result was replicated across the different temperatures: by taking into account differences in mosquito survival, our EIP90 estimates are higher than those from the logistic method”.

To what extent may the difference in the variation in the EIP be due to the different model types (see also comment 2)?

This is a very reasonable point, which we did not previously cover sufficiently in the manuscript, and we thank the reviewer for highlighting this. Since the EIP is an unobserved quantity, a model is needed to estimate it, and different models will yield different estimates. Previous attempts to model the EIP simply fitted an arbitrary function to the data, which did not account for the mechanisms leading to the rise in sporozoite prevalence. By fitting a flexible function they were able to obtain a close fit to the data which was interpreted as meaning there is low variation in the EIP. Our model includes the biological processes thought to be most responsible for the observed parasite population dynamics. Using a model that includes these processes leads to an alternative EIP estimate with greater variability.

An important result of fitting a mechanistic model is that it allows us to determine whether further processes might be needed to explain the data. Indeed, we found that at higher temperatures, the processes and data we include do not fully explain the data, which we discuss in the second paragraph in the discussion (lines 628 – 707):

“The influence of parasite-induced mosquito mortality is still a source of debate and likely to vary depending on the parasite-vector system [26,37]. Nonetheless, accounting for potential differences in mortality is essential to avoid bias in parameter estimates: our estimates of the human-to-mosquito transmission probability, for example, were higher than those estimated by the logistic model. By modelling mosquito infection and survival in a single framework we could estimate malaria infection induced differences in mosquito survival, whilst accounting for mosquitoes that do not develop oocysts within the infectious blood fed group. At higher temperatures, we found that the modelled differences in mosquito survival only partly explained the sporozoite prevalence data. Another study observed no difference in the survivorship of wild caught *A. gambiae* mosquitoes due to malaria infection, yet *P. falciparum* detection did decline over time [50]. So, it is possible that mosquito survivorship was not completely responsible for these observed declines and alternative explanations, such as sporozoite loss during sugar-feeding [51,52] or sporozoite mortality could require further investigation.”.

To address further the point about potentially missing mechanisms, we now include the following in our discussion (immediately following the above text; lines 707 – 710):

“Including other such mechanisms in our model could lead to alternative estimates of the EIP. This means that it is possible that the differences between our EIP estimates and those previous could partly be because important, yet unknown, mechanisms were not included in our model”.

2. What informs the heterogeneity found by the model? Is it heterogeneity in the data, the construction of the model that allows for distributions e.g. in parasite development times, or is it due to the choice of the priors? If it is informed by heterogeneity in the data, then in this reviewer’s opinion the manuscript could be improved by including a table or figure (maybe in the supplement) that shows the heterogeneity in the data and compares it to the heterogeneity in the two models. In this case, it might also be beneficial to show (e.g. in a supplementary figure) that the heterogeneity in their model does not depend on the choice of the priors but can also be found using uninformative priors instead of weakly informative priors.

We agree the reasons for the heterogeneity found by the model is an important question. The heterogeneity in our results is informed by the data and model choice (see our answer to Major comment #1 for why this is necessary, and we believe an argument for using a mechanistic model).

Thank you for your suggestion regarding the table, unfortunately, EIP cannot be directly estimated at the individual mosquito-level from these data as mosquitoes are killed during the dissection process (and therefore we cannot retest the same individual). This precludes us from directly comparing the data and model heterogeneity. Nonetheless, the raw data shows a wide range of EIP values as indicated by the slope of the increase in sporozoite prevalence (presented in Figure 2), which indicated not all mosquitoes develop sporozoites at the same time. The new model is broadly able to capture the upward trend in sporozoite prevalence as illustrated in Figure 3 (particularly at low temperatures) and Figure S7. This generates greater variability in the EIP estimates, as demonstrated by the difference between EIP_10_ and EIP_90_ in Figure 4A. Comparable prediction using the logistic function can be found in Figure S11. Here you will see the logistic function generates, on average, less heterogeneity, in part because it makes the assumption that all mosquitoes have developed sporozoites by the peak in sporozoite prevalence. To illustrate this point and highlight the differences in heterogeneity between the different models in Figure 4a we have included the EIP_10_, EIP_50_ and EIP_90_ estimates for each model.

We do not believe the modelled heterogeneity is due to the weakly informative priors, because, in general, our priors were substantially different from the posteriors (see Figure S4). For more details on prior choice please see our response to the point made by reviewer #2 regarding our choice of priors.

3. In the discussion (lines 516-517), the authors claim to have shown “predictive accuracy”. In this reviewer’s opinion they have shown that their model is a visually better fit to the data than a logistic model, which has less flexibility and was fit to only a part of the data. It is not clear in which way they have shown “predictive accuracy”.

We agree that we have not quantified “predictive accuracy”. We are not trying to directly compare which model fits the points best as the models are very different: the logistic model simply fits a line through the points, whereas our model attempts to capture the processes involved. We have consequently removed reference to predictive accuracy: lines 618 – 620 have been updated accordingly:

“To illustrate its use, we fitted mSOS to experimental data and demonstrated it can produce a reasonable visual fit to the data across a range of experimental protocols”.

Minor comments:
4. Equation (2.08) (lines 296-299), should E(.) be replaced by En(.)?

You are correct, thanks for catching this: this has been amended.

5. The authors state their model is mechanistic, but it seems to this reviewer that it is describing features of the biological process (e.g., the time for the parasite to reach the next development stage) rather than mechanisms causing these features.

You are right that we do not model the individual biochemical processes that result in (say) an oocyst to transition into a sporozoite. This is due to the current availability of experimental data in this system. Should such data become available in the future, we would jump at the opportunity to use it to further refine our model. We do think that it is worth reiterating though that our model does explicitly include a number of larger-scale biological features: it models the individual parasites within individual mosquitoes, all of which dynamically evolve; it also models mosquito survival. These processes have not been considered in the previous attempts to fit sporozoite prevalence, which just fit an arbitrary function to data. Because of this, we’d argue that our model can be described as “mechanistic”.

Reviewer #2: Stopard et al. developed a mechanistic mathematical model of malaria parasite sporogony and estimated the parameters of the model by fitting the model to a multitude of experimental data. They found their mechanistic model predicted different EIPs from previously reported values and what a statistical model (e.g. logistic model) predicted. In addition, they also examined the impact of temperature on EIP. Controlling malaria transmission from human to mosquito represents one of the crucial approaches to achieving malaria elimination. Given that a quantitative understanding of how different biological factors influence the probability of successful transmission is still very limited, this study represents a very good effort to fill the knowledge gap using data-based modelling approaches. The manuscript is well written, and the models and methods are proposed rigorously and presented clearly. Therefore, I think this work will be suitable for PLoS Computational Biology after addressing the following comments.

We thank the reviewer for their useful comments and kind words. We agree that a quantitative understanding of the different biological factors affecting transmission and the development of the *Plasmodium* parasite within the mosquito is limited, thank you for your insights into this topic. In what follows, we will hopefully address each comment.

1. Lines 177-180: provide some rationales for the assumptions of which are time-dependent and which are not.

Those corresponding lines are about temperature dependence, so we address this comment as if so.

We allowed mosquito survival (in the laboratory), the pre-oocyst development rate and human-to-mosquito transmission probability to be temperature-dependent based on the results of previous studies. It is possible that the oocyst to sporozoite development rate is temperature-dependent but given the lack of time-points of oocyst dissection, we could not assess this, and therefore relied on the existing literature which suggests the pre-oocyst stages are the most temperature-sensitive. These studies have been referenced in the main text: for clarity we have adjusted the wording of lines 213 – 218 to,

“Experimental evidence indicates that the pre-oocyst life stages are the most temperature sensitive [41,42], and temperature affects both the human-to-mosquito transmission probability [27] and laboratory mosquito survival [14]. We therefore allowed these processes to be influenced by temperature. The development time from oocyst to sporozoites and the mean parasite load among infected mosquitoes was assumed to be independent of temperature”.

Thanks for highlighting the need for more detail here.

2. For model fitting, are there any correlations between model parameters identified based on posterior samples? if there are, how do they influence the parameter estimates reported in the main text?

This is a good point, and we now include the pairwise plot of posterior samples for the “all temperature model” in Figure R1 (now included in the supplementary information - Figure S4). Lines 441 to 444 have been updated to:

“We visually examined the trace plots (Fig S3), pairwise plots of the MCMC parameter values (Fig S4) and difference between prior and posterior distributions (Fig S5)”.

Within the hierarchical all temperature model there are correlations between the G to O shape parameter (α_GO_) and the parameters describing the relationship between temperature and the G to O rate parameter (m_β_ and c_β_). Similarly, there is correlation between the O to S gamma distribution shape parameter (α_OS_) and the O to S gamma distribution rate parameter (β_OS_). The mean of a gamma distributions is given by $\frac{shape}{rate}$ . Consequently, a linear line of $shape=rate$ will give the same mean, and likely (because variances are typically harder to cleanly estimate), a similar fit to the data. Because of this, the posterior samples of shape and rate parameters are linearly correlated.

I also notice that some priors (in Table S1) are Gaussian with an SD of 2.5, e.g. alpha_OS, alpha_GO, mu, k, a and b, etc. It is fine for mu, k, a and b because the priors look less informative compared to the posteriors. However, for alpha_OS and alpha_GO, the priors may be very influential. So could the authors provide some justifications for the choice of SD = 2.5 as “prior knowledge”? If 2.5 was chosen arbitrarily, the authors should relax the constraint, say using a larger SD, and examine if the estimates are robust to the choice of the SD.

You are correct that the choice of SD = 2.5 is somewhat arbitrary, but because of the nature of how the shape and rate parameters jointly determine the mean, it is not a binding constraint. We illustrate this now using your helpful comment about an additional experiment. Specifically, we reran the MCMC with SD = 4.5 on the α_GO_ and α_OS_ parameters. Figure R2 shows there are small differences in the posterior distributions of the gamma distribution parameters when using a SD of 4.5 on shape parameters. Figure R3, however, indicates the modelled sporozoite prevalence is only very slightly altered by changing the SD on these priors. As noted in response to question 2, the mean of the gamma distribution is co-determined by both the shape and rate parameters and increasing the SD on the shape parameter priors from 2.5 to 4.5 only leads to minor changes in the mean parasite development time from G to O from 11.76 to 11.69 days (posterior mean). It also leads only to small changes in the difference between the 5% and 95% quantiles of this quantity (from 0.53 to 0.57 days). We believe this impact is minimal and have therefore kept the SD as 2.5 but thank the reviewer for the suggestion.

3. When comparing the waiting time before sporozoite positive between model prediction and experimental observation, i.e. lines 402-404, it would be more informative to report a comparison at a comparable prevalence such that the model prediction is more relevant to the “reailty”. For example, instead of only examining EIP10 or higher, predictions of EIP1, EIP2 and EIP5 should also be included in the results.

Thank you for highlighting this. We believe there is more uncertainty in the lower and upper modelled sporozoite prevalence estimates and have therefore used the time at which sporozoite prevalence reached 5% of all dissected mosquitoes. Please note that when comparing the variability, we assess the range between EIP_10_ and EIP_90_, which we have more confidence in. Nonetheless, we agree it is a good idea to report a more comparable modelled sporozoite prevalence and have updated lines 475 – 480 to,

“In the raw data, sporozoite-positive mosquitoes were first observed on day 10 when the sporozoite prevalence was 1.4%; our model estimates the appearance of sporozoite-positive mosquitoes earlier, with the model estimating a sporozoite prevalence of 5% at 9.0 days (CI: 8.8–9.2 days) (Fig 2c). The time required for sporozoites to appear varied between mosquitoes, and, only after 15.9 days (CI: 14.2–16.0 days) did the modelled sporozoite prevalence peak at 63% (CI: 61–65%)”.

Minor comments:
Line 23: EIP10 and EIP90 (and EIP50) were not defined in the abstract.

We agree this should be defined and have updated the abstract lines 17 – 19 accordingly,

“The EIP is typically estimated as the time for a given percentile, $x$, of infected mosquitoes to develop salivary gland sporozoites (the infectious parasite life stage), which is denoted by EIP_x_.”

Line 374: provide Stan version number

Thank you, the Stan and R version numbers used have now been included on line 436.

Reviewer #3: This manuscript by Stopard and colleagues describes the development of a novel within-vector mechanistic model of a key step in the transmission process of the malaria parasite. The analysis is thorough, well described and the writing of the manuscript is exceptional throughout. While I do have some specific queries about some parts of the analysis and how it is presented, overall I believe the stud is of high quality and should be published.

Thank you taking the time to review our manuscript, your kind words and support for our manuscript. We really appreciate your useful insights, feedback and queries, which we will hopefully respond to in detail below.

Major comments:

This study fits the model to data from four pre-existing studies. It is not clear in this manuscript why these four studies were chosen. Presumably there are many more studies that have measured EIP previously. Classically I would have expected either a thorough systematic literature search to identify all possible data sources or a strong justification of why a particular study / small group of studies was chosen for this analysis, but currently there is neither.

We limited the model fitting to a small number of studies for the following reasons:

1. Within this study we are primarily interested in demonstrating the use of this model in highly controlled conditions and membrane feeding assays are sensitive to small changes in experimental protocol.
2. Detailed data is required to fit the model which requires:
   a. the dissection of mosquitoes for different parasite life stages (oocyst and sporozoite) over a wide range of days
   b. mosquito survival data (both infected and uninfected)

Initially, we conducted an (unsystematic) review to identify potential data sources, but given the specific requirements of the data (we noted lots of dissection data is limited to a small number of days which doesn’t allow characterisation of the EIP) and the numerous further experimental (and potentially not quantified) variables that would need to be considered, we decided to limit the datasets used. We agree that in the future it would be useful to conduct a more systematic review, however. Indeed, we believe that this sort of resource could be of use to both modellers and experimentalists. To clarify this we have added the following section to the end of the introduction ( lines 135 to 140):

“We fit mSOS to laboratory data and present new EIP estimates which contrast with those estimated using the predominant approach in the literature. This is intended to illustrate the application and implications of using a mechanistic model of sporogony, and not to summarise current understanding of the EIP, which would require a wider systematic review”.

While the model has clear strengths for representing and predicting various transmission processes, I did find the suitability of the model to represent changes in response to temperature changes less convincing compared to other results. The data used span a limited range of temperatures (particularly excluding extreme low or high values), a step of the analysis fixes a linear development rate (line 346) based on the analysis of data from a limited range of temperatures and the fit of the model shows increasing disparity in shape with higher temperature (Figure 3). These are fairly minor issues in themselves, but in the discussion mSOS is explicitly compared to models designed to explore the thermal limits of transmission (line 541). While the results are caveated and some development is suggested, I just don’t see this particular application of mSOS as having been conclusively proven in this manuscript.

Thank you for your detailed insights here. We agree that we do not probe the actual thermal limits of transmission and that fixing a linear development rate limits the ability to explore the thermal limits of transmission. As you state this is due to the lack of extreme high and low temperature data in the experimental data. Nonetheless, our individual fits to each temperature separately suggested that a linear relationship for those parameters we modelled as temperature-dependent was appropriate across the range of temperatures in the dataset.

We are aware of studies that model the thermal responses as either quadratic or Brière functions (1,2) and have also run our model where we fit the relationship between temperature and the human-to-mosquito transmission probability to be logistic-quadratic and the relationship between temperature and the inoculant (G) to oocyst (O) development rate to be quadratic. This results in marginal differences in the modelled sporozoite prevalence (Figure R4). We didn’t present these in the manuscript, however, as we are wary of overfitting our dataset: given further data across a wider range of temperatures, we would definitely like to revisit these ideas. Nonetheless, we agree that we do not fully capture this detail in the manuscript and have updated this paragraph in the discussion (lines 711 – 744).

“Temperature is an important predictor of *P. falciparum* prevalence among humans: its impact is, however, often non-linear and location-specific [53–55]. Laboratory data are used to determine temperature-driven variation in the EIP, which is commonly used in the prediction of the spatiotemporal limits and endemicity of malaria transmission [32,56,57]. Over the temperature range investigated, we determined that a linear relationship between temperature and parasite development rate was a reasonable approximation. But, we recognise that the data does not capture the thermal limits of sporogony, and, to handle these would likely require including non-linear relationships (for example see [32]). Our EIP estimates qualitatively match those of [15], which determined that the EIP falls with increases in temperature, and that the incremental change is greatest at lower temperatures. In a semi-field setting, increases in temperature, due to the interaction between deforestation and altitude, decreased the EIP of wild *P. falciparum* [19]. But very little field data exists, and further experimental studies across the whole temperature range of the disease with natural vector-parasite combinations are required to capture the full relationship between temperature and EIP. To more accurately simulate the EIP in the field will require consideration of the impact of the time of biting [58], diurnal temperature fluctuations [59] and mosquito resting location on the temperatures parasites are exposed to during different life stages. The degree to which the EIP of locally adapted strains differ to the laboratory strains must also be considered [60]. Current estimates indicate climate change has the potential to change the number of people at risk of malaria [57,61,62], though there is uncertainty due to the complexity of how the parasite and mosquito will interact with the local changing environment in the long term. Characterising spatiotemporal heterogeneity in the EIP in the field is thus critical to assess our current understanding of malaria and its control as well as the changing risk of malaria resulting from the interactions between climatic, land-use [63] and socio-economic factors [64]”.

Minor comments:

Approximation of T(GS) (line 218) – is this just for studies that didn’t observe intermediate parasite forms? Worth stating if so, also any idea how good this approximation is?

The approximation of T_GS_ is to estimate the CDF of the sum of the two gamma distributed development times, from G to O and O to S. An analytical solution for the CDF is not available (hence the approximation using a single gamma distribution with the same mean and variance, see (3)).

We did some exploration of this approximation and found it to be reasonable across the range of parameters we surveyed. For example, in Figure R5, we plot the approximate PDF (blue line) against the true PDF (orange line) using the mean posterior parameter values at 27°C. This shows there are very few small differences at this parameter set. (Unfortunately, the functions in Mathematica which could produce an analytic PDF were not available in Stan: hence our reason for using the approximation).

Study random effect- defining unique studies as different study/ temperature combinations seems strange when you are also trying to estimate the effect of temperature alone. Might be better to restrict this to just unique study combinations as I would expect between study variation to be much greater than within study but at different temperature variation.

This is a good point, and we agree that between-study variation would likely be much greater than the within-study variation. Our model allows us to capture both sorts of variation but, as you say, an alternative way to handle this would be to explicitly partition these two sources. We chose not to do this here since we did not have a great deal of nested variation (i.e. observations within individual temperature experiments within studies). Any future work involving pooling large numbers of observations across multiple study types would likely merit the sort of partitioning that your comment suggests, and we thank you for this useful suggestion.

Further changes

1. Author affiliation updated.
2. There was a couple of typos in the abstract results values, these have been updated to match the correct in text values.
3. Author summary (lines 35 to 47) updated to focus on the EIP,

*“Anopheles* mosquitoes become infected with malaria-causing parasites when blood feeding on an infectious host. The parasites then reproduce via a number of life stages, which begin in the mosquito gut and end in the salivary glands, where the newly formed infectious parasites can be transmitted to another host the next time a mosquito blood-feeds. This delay in the mosquito becoming infectious, known as the extrinsic incubation period (EIP), is important in determining whether a mosquito is able to transmit malaria. The large variability in parasite numbers and development times that exists between mosquitoes, environments and parasites mean that estimating the EIP is difficult. Here, we introduce a mathematical model of the population dynamics of the mosquito life stages of the parasite, which mimics key characteristics of the biology. We show that the model’s parameters can be fit so that its predictions correspond with experimental observations. Our work is a step towards a realistic model of within-mosquito parasite dynamics, which we hope can be applied to help understand heterogeneity in malaria transmission”.

4. Introduction lines 121 – 124 made more concise,

“To date, the relationship between temperature and the EIP has been modelled using a degree-day model, which parameterises the total amount of heat required to complete sporogony [30], or polynomial (quadratic or Brière) functions [31,32]”.

5. Added the recently published reference to line 127 - Childs LM, Prosper OF. The impact of within-vector parasite development on the extrinsic incubation period. R Soc Open Sci. 2020 Nov 16;7(10):192173.
6. Lines 319 – 320 updated to: “Mosquitoes die throughout the course of the experiments, which we explicitly model using a Cox regression model.”
7. Lines 750 – 752 updated to: “Indeed, to parameterise mSOS, we collated data from four previously published experimental infections that produced data with high temporal resolution. To characterise parasite development, it would be useful if future studies dissected mosquitoes across a greater range of times post infection”.
8. Updates lines 762 – 771 to: “The work shows a possible mechanism for how higher parasite loads within a mosquito might decrease the EIP, which could potentially have important epidemiological consequences, since it indicates that onwards transmission may be more efficient from more infectious people. This relationship has also recently been considered in another modelling study [35], which found that the impact of parasite load on the EIP of *Plasmodium* *berghei* is non-linear. Other processes may be operating: emerging experimental evidence hints that a decrease in resource availability per parasite (at high parasite loads), as determined by the parasite load and the number of times the mosquito blood-feeds, may decrease the parasite development rate [49,68]. So, to fully capture this relationship may require the incorporation of density-dependent mechanisms”.
9. Lines 779 – 781 updated: “Our model, or ones like it, could be extended to incorporate more fine scale characteristics of parasite ecology, but to do so in a principled manner requires more fine scale data”.
10. The use of the word “Figure” has been updated to “Fig” as recommended for the PLOS format.
11. Figure titles and captions have been moved the appropriate place in text.
12. References have been put into the PLOS format.
13. The document has been separated into separate documents for the manuscript text and supplementary information.
14. References to the updated supplementary information have been updated to represent the correct numbering now the supplementary information is an independent document.

References

1.	Mordecai EA, Paaijmans KP, Johnson LR, Balzer C, Ben-Horin T, Moor E, et al. Optimal temperature for malaria transmission is dramatically lower than previously predicted. Ecol Lett. 2012 Oct 11;16(1):22–30.

2.	Villena OC, Ryan SJ, Murdock CC, Johnson LR. Temperature impacts the transmission of malaria parasites by Anopheles gambiae and Anopheles stephensi mosquitoes. bioRxiv. 2020 Jan 1;2020.07.08.194472.

3.	Stewart T, Strijbosch LWG, Moors H, Batenburg P van. A simple approximation to the convolution of gamma distributions. 2007.

**Figure R1** Pairwise comparison of parameter estimates from the all temperature model fit (now included in manuscript as Figure S4).

**Figure R2** The difference between posterior distributions when changing the standard deviation (SD) on the α_GO_ and α_OS_ normal priors set from 2.5 to 4.5.

**Figure R3** Changing the standard deviation (SD) on the α_GO_ and α_OS_ normal priors set from 2.5 to 4.5 has very little impact on the modelled sporozoite prevalence.

**Figure R4** Sporozoite prevalence when the relationships between temperature and the human-to-mosquito transmission probability and temperature and the inoculant (G) to oocyst (O) development rate are modelled as logistic-quadratic and quadratic respectively.

Days

Probability density

**Figure R5** Difference between the approximate PDF (gamma distribution with the same mean and variance, blue line) and analytical PDF (orange line) of the sum of two gamma distributed random variables. Parameter values were chosen to match those of the fitted mean posterior values at 27°C.
